# Effects of Peanut Rust Disease (*Puccinia arachidis* Speg.) on Agricultural Production: Current Control Strategies and Progress in Breeding for Resistance

**DOI:** 10.3390/genes15010102

**Published:** 2024-01-15

**Authors:** Yu You, Junhua Liao, Zemin He, Muhammad Khurshid, Chaohuan Wang, Zhenzhen Zhang, Jinxiong Mao, Youlin Xia

**Affiliations:** 1 Nanchong Academy of Agricultural Sciences, Nanchong 637000, China; yuyoujtsd@163.com (Y.Y.); liaojunh@hotmail.com (J.L.); hezeminyx@hotmail.com (Z.H.); wangchaohuan12@163.com (C.W.); zhangzz1103@163.com (Z.Z.); maojinx1973@163.com (J.M.); 2 School of Biochemistry and Biotechnology, University of the Punjab, Lahore P.O. Box 54590, Pakistan; khurshid.ibb@pu.edu.pk

**Keywords:** peanut rust, resistance resource, resistance gene, rust-resistant breeding, molecular breeding technique

## Abstract

Peanuts play a pivotal role as an economic crop on a global scale, serving as a primary source of both edible oil and protein. Peanut rust (*Puccinia arachidis* Speg.) disease constitutes a significant global biotic stress, representing a substantial economic threat to the peanut industry by inducing noteworthy reductions in seed yields and compromising oil quality. This comprehensive review delves into the distinctive characteristics and detrimental symptoms associated with peanut rust, scrutinizing its epidemiology and the control strategies that are currently implemented. Notably, host resistance emerges as the most favored strategy due to its potential to surmount the limitations inherent in other approaches. The review further considers the recent advancements in peanut rust resistance breeding, integrating the use of molecular marker technology and the identification of rust resistance genes. Our findings indicate that the ongoing refinement of control strategies, especially through the development and application of immune or highly resistant peanut varieties, will have a profound impact on the global peanut industry.

## 1. Introduction

Peanut (*Arachis hypogaea* L.), an allotetraploid (AABB, 2n = 4x = 40) originating from South America, constitutes an important feed, food, and oilseed crop due to its high nutritional and economic value worldwide. Cultivated across 114 tropical and subtropical countries, this crop spans an extensive area of 32.72 million hectares (M ha), yielding an annual production of 53.93 million tons (MT) and a productivity rate of 1648 kg/hectare [1]. The three leading planting and producing countries, India, China, and Nigeria, collectively account for 45.81% and 61.58% of the global area and production, respectively. China, with a production of 18.36 million tons (MT), is the world’s largest peanut producer. However, India has the largest cultivation area, covering 5.97 M ha, while China’s area is 4.75 M ha [1]. Despite growing interest and demand, peanut crops face substantial challenges in terms of quality and yield, primarily due to various biotic and abiotic stresses—notably, fungal diseases.

Peanut rust (*Puccinia arachidis* Speg.) is a prevalent fungal disease that predominantly targets peanut leaves but can also affect petioles, support leaves, stems, and other plant parts. Several names were given to the pathogen causing peanut rust since the first report from Suriname in 1827 [2]. Ultimately recognized as a distinct species by Spegazzini in 1976, it was officially named *P. arachidis* [3]. This widespread rust disease is prevalent wherever peanuts are cultivated [4], and it thrives in warm and humid conditions, facilitating rapid spread by way of repeated uredospore infection cycles [5]. The impact of peanut rust on the peanut industry is severe, significantly hindering its development. Infected peanut plants exhibit various symptoms of damage during the growing season, including early pod maturity, increased pod senescence, reduced seed size, and decreased oil content [6]. Research conducted by Mondal and Badigannavar indicates that rust disease can lead to yield reductions of up to 57% in susceptible genotypes such as Robut 33-1, resulting in substantial economic losses [7].

Given the significant nutritional and economic value of peanuts, a range of strategies have been employed to combat peanut rust in peanut production. These encompass cultural, chemical, and biological control methods and host resistance. However, it is crucial to weigh up the drawbacks of the chemical and cultural methods, which not only increase production costs but also pose risks to food safety and the environment. In contrast, host resistance and the development of disease-resistant cultivars stand out as the most sustainable strategies against peanut rust [6]. Consequently, breeding peanut varieties that are resistant to rust has become a focal point for private, national, and international research institutions. While substantial strides have been made in breeding rust-resistant varieties and lines, the creation and deployment of immune or highly resistant varieties that are adaptable to diverse production areas remain vital for all peanut-growing nations around the world.

This review aims to showcase the progress that has been made in this field to inspire and galvanize further research and innovation in countering this challenging disease. By highlighting the progress achieved, we hope to instigate a collective effort towards the development of rust-resistant peanut varieties, anticipating the positive and transformative impact of such research on the industry.

## 2. Characteristics and Disease Symptoms of Peanut Rust

Peanut rust (*P. arachidis* Speg.) is taxonomically classified within the phylum *Basidiomycota*, subphylum *Pucciniomycotina*, class *Pucciniomycetes*, family *Pucciniaceae*, and genus *Puccinia* [8,9]. As a fungal disease positioned within the realm of higher fungi, it has undergone extensive scrutiny to identify its characteristics and detrimental symptoms. Understanding the biology and pathogenicity of peanut rust is of paramount importance, serving as the bedrock for the formulation of effective management and control strategies. Researchers have invested considerable efforts in exploring the disease’s life cycle, modes of infection, and factors influencing its spread and severity. The wealth of knowledge derived from these investigations plays a pivotal role in crafting sustainable and efficient approaches to combatting peanut rust, thereby mitigating its impact on peanut cultivation globally.

### 2.1. Life Cycle of Peanut Rust

**Figure 1:**
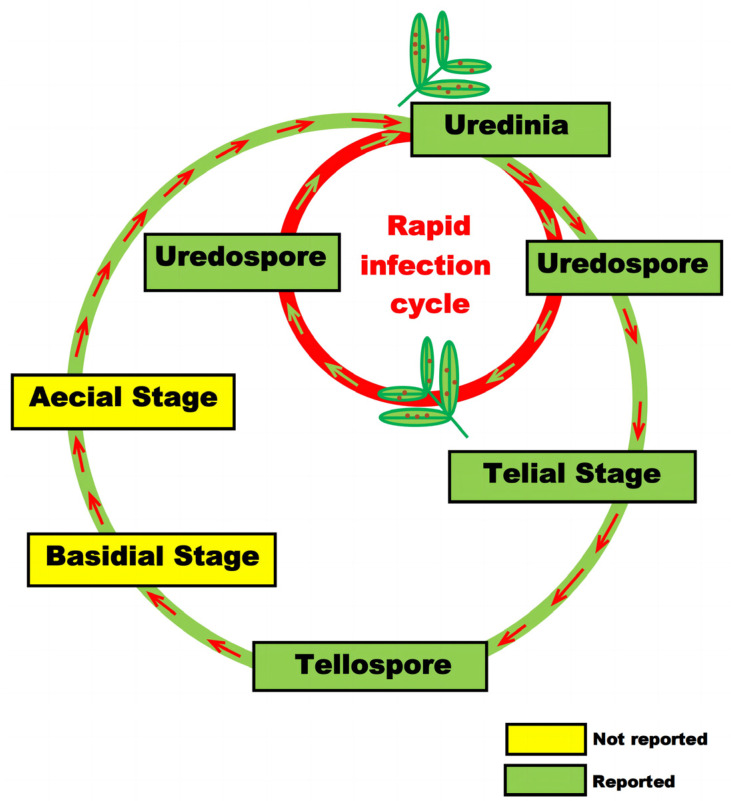
Life cycle of peanut rust (*P. arachidis* Speg.).

The sexual life cycle of peanut rust is a complex process involving transitions between haploid and dikaryotic stages, encompassing five distinct spore stages: the spermagonium (haploid), aecium (dikaryotic), uredium (dikaryotic), telium (dikaryotic), and basidium (dikaryotic/diploid) [5] (Figure 1). Notably, the spermagonium and aecium stages occur in an alternate host, whereas the uredium, telium, and basidium stages perpetuate in the primary host. The formation of the dikaryotic mycelium results from plasmogamy, a fusion between two compatible spermatids and receptive hyphae.

The presence of the aecial form in peanut rust remains unclear given the absence of an alternate host. However, the uredial form predominantly sustains the disease in nature [10]. Uredospores, characterized by their obovate, ovate–oblong, or broadly clavate shape, infect peanut leaves and give rise to uredinia. These uredinia are hypophyllous, subepidermal, ellipsoidal, or oblong structures, each housing numerous pedicellate uredospores [10].

The sexual life cycle of peanut rust encompasses five spore stages: spermagonium (haploid), aecium (dikaryotic), uredium (dikaryotic), telium (dikaryotic), and basidium (dikaryotic/diploid). The spermagonium and aecium stages occur in an alternate host, while the uredium, telium, and basidium stages perpetuate in the primary host. However, it remains unclear whether the aecial form and basidial form exist in peanut rust due to the lack of an alternate host.

Upon maturity, the uredinia transform, turning dark cinnamon brown and releasing a multitude of uredospores upon bursting. Under natural conditions, these nascent uredospores initiate several infection cycles in susceptible hosts. The tibial stage, formed from uredospores under low temperatures and nutrient stress, is not commonly observed in peanut rust [5]; however, instances have been reported in wild peanut leaves in Florida, Brazil, and Central America, as well as in cultivated peanut fields in Karnataka, India, and Gadar [3,10].

Telia, which contain numerous teliospores, are part of the life cycle, but their presence has been minimally reported worldwide. Consequently, further research is imperative to elucidate the existence and function of teliospores [3,10,11]. Teliospores and basidia contribute to sexual spores in the peanut rust life cycle, fostering genetic sequence variability among different isolates and potentially leading to the evolution of new pathotypes or races [10].

Importantly, due to the absence of the basidium, occurrences of karyogamy and successive meiosis in the basidium have not been reported, and the emergence of new variants or races of peanut rust has rarely been observed to date [10]. The intricate and nuanced aspects of the peanut rust life cycle underscore the necessity for ongoing research to deepen our understanding of this pathogen and enhance strategies for its management.

### 2.2. Infection Process

The infection process of peanut rust plays a pivotal role in the progression of the disease. Following the germination of the uredospores, a singular unbranched germ tube emerges from one of the equatorial germ pores in its wall [12]. Notably, the germination of uredospores in dense patches and clumps is impeded by a higher concentration of methyl cis-3,4-dimethoxycinnamate [13]. The germ tube extends along the intercellular grooves of the peanut leaf’s surface until it establishes direct contact with the stomata. Upon contact, the tip of the germ tube undergoes swelling, giving rise to a thin-walled ellipsoidal appressorium of approximately the same size as the emerging spore [14].

The processes of germ tube elongation and appressorium formation are typically completed within 12 h of inoculation in susceptible genotypes. A thin cross wall forms between the appressorium and the germ tube, encapsulating the dense cytoplasm within the appressorium [15]. Subsequently, a narrow infection peg emerges from the appressorium, traversing the stomatal apertures and swelling to form a vesicle in the substomatal chamber. Within 24 h, several infection hyphae (dikaryotic) typically develop from the substomatal vesicle, forming simple knob-like haustoria in adjacent mesophyll cells.

**Figure 2:**
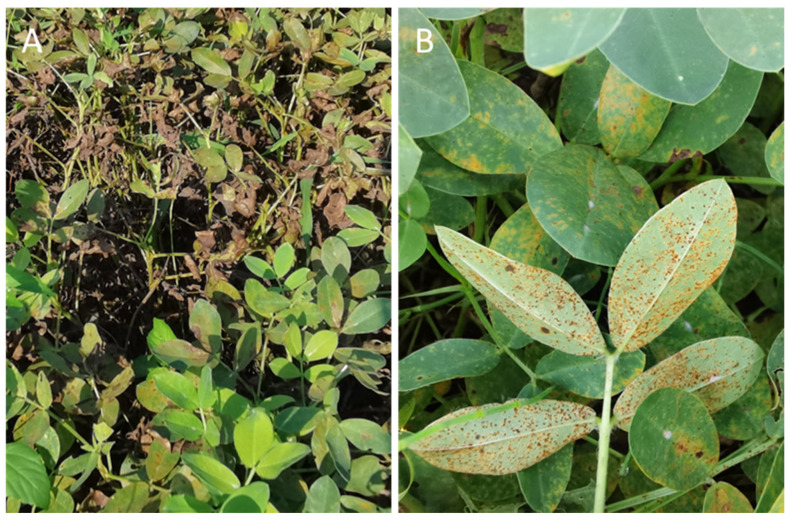
Field phenotype of plants infected with peanut rust disease. (**A**) The harm caused to peanut field production by peanut rust disease; (**B**) typical symptoms of rust disease on peanut leaves.

During this phase, the pathogen employs hydrolytic enzymes, including cellulases, glucanases, and proteinases, which play a crucial role in dissolving cell walls and plasma membranes, facilitating intercellular infection. These infection foci evolve into chloronemic flecks, eventually transforming into reddish-brown or orange pustules, known as uredinia, on the lower leaf surfaces (Figure 2).

Ultrastructure studies utilizing scanning electron microscopy have revealed differences in spore reactions on the lower leaf surfaces of two peanut genotypes: *Arachis stenosperma* V10309 (resistant) and *A. hypogaea* cv. IAC-Tatu (susceptible) [16]. In the susceptible genotype, the germ tube elongates sufficiently to successfully enter the stomata, resulting in a successful intercellular infection within 72 h of inoculation [5]. In contrast, the resistant genotype exhibits distinct responses during the infection process, highlighting the intricate dynamics involved in the interaction between the peanut plant and the rust pathogen.

### 2.3. Disease Symptoms of Peanut Rust

Peanut rust primarily targets leaves, but it can also affect petioles and stems. Identifying the disease is quite straightforward as visible rust spores appear on the peanut plant. Symptoms of peanut rust typically arise around 8–10 days after the infection. It all begins with whitish flecks appearing on the lower leaf surface (abaxial). Within a day, yellowish flecks start showing up on the upper leaf surface (adaxial); meanwhile, on the lower side, orange–red or brownish pustules (known as uredinia) start forming. Approximately two days after these symptoms emerge, raised pustules become visible. They often take on a circular to elliptical shape, measuring 0.3–2.0 mm in diameter. These pustules exhibit elliptical or circular uredospores; these are initially dark orange but mature into a cinnamon brown color [17] (Figure 2).

While pustules might occasionally appear on the upper leaf surface, they are not as plentiful as those on the lower side. When necrosis develops around these pustules, it can cause lesions to merge, ultimately resulting in leaf defoliation. Peanut rust typically spreads outwards in a radiating pattern from a single spot in the field. Under warm and humid weather conditions, the affected area can expand rapidly (Figure 2). Recognizing these distinctive symptoms early on is pivotal for promptly implementing management strategies to curtail the disease’s impact on peanut crops.

## 3. Epidemiology and Control Strategies for Peanut Rust

Epidemiological studies are pivotal in advancing our understanding of the occurrence and distribution of peanut rust. With their meticulous analyses of the factors influencing the spread and severity of the disease, these studies enable us to forecast the epidemic trend. These predictive capabilities form a robust foundation for implementing efficient strategies in controlling and preventing peanut rust.

### 3.1. Epidemiology

Uredospores play a crucial role in the persistence and dissemination of the peanut rust pathogen across seasons [18]. Their dispersal involves various mechanisms, such as rainfall, wind, and adherence to plant materials [19]. It is noteworthy that uredospores are airborne and not internally seed-borne spores [20,21].

The epidemiology of peanut rust is profoundly shaped by environmental conditions. Studies indicate that the disease thrives under sustained high humidity (>78%) and warm temperatures (20–30 °C) [5,20]. Conversely, the disease exhibits sluggish progression when temperatures drop below 10 °C or exceed 35 °C [22]. Uredospores can maintain viability for up to 20 days at field temperatures ranging from 25 °C to 28 °C, with an optimal germination temperature of 25 °C [18]. The controlled manipulation of humidity and temperature in experimental environments proves instrumental in regulating the rate of inoculum accumulation.

Environmental variables, including humidity, temperature, and wind speed and direction, wield a substantial influence on the distribution, infection, and development of airborne fungi, including peanut rust [23]. The prudent management of wind speeds and directions under specific production conditions can effectively curtail the buildup and spread of the inoculum.

The epidemiology of peanut rust is further complicated by the host genotype. Distinct peanut genotypes may exhibit varying levels of susceptibility to the disease under diverse temperature conditions [22]. Light rain showers are markedly more conducive to disease dispersal than heavy showers as the latter significantly diminish the pore content in the canopy [24]. Early sowing practices can mitigate the severity of peanut rust during the summer, while late sowing may assist in reducing disease incidence during the rainy season [25]. Additionally, levels of spore trapping on the plant canopy tend to be lower in the evening than in the morning, and the overlapping of crop seasons contributes to continuous inoculum buildup and the aerial propagation of uredospores [5,25].

### 3.2. Control Strategies for Peanut Rust

Controlling peanut rust is of paramount importance for mitigating the disease’s impact and enhancing the efficiency of peanut production. Control strategies can be categorized into four main approaches:

(I) Cultural control: quarantine stations play a crucial role in this strategy. Stringent phytosanitary inspections are imposed to regulate the movement of peanut materials across regions, preventing the introduction and spread of peanut rust to new areas. Employing crop rotation with cereals and other non-host species proves effective in limiting the spread of peanut rust in susceptible regions [14]. Cultural control also entails the careful consideration of suitable sowing dates, the appropriate planting density, weeds, fertilizers, and water management, along with the implementation of fallow periods [26]. The proper removal of diseased residues after the peanut harvest is imperative for preventing further spread in farmers’ fields.

(II) Chemical control: in cases of unexpected and severe infection or outbreaks where cultural control measures are insufficient, it becomes necessary to resort to chemical control. Fungicides such as carbendazim and chlorothalonil are commonly employed to reduce the incidence of peanut rust, with chlorothalonil being reported as the most effective fungicide for controlling peanut rust [26,27]. However, chemical control has its drawbacks, including high costs, potential environmental and health hazards, and the risk of resistance buildup in pathogenic strains, making it less than ideal for long-term management.

(III) Biological control: biocontrol agents, including Acremonium obclavatum [28], Bacillus subtilis AF 1 [29], Verticillium lecanii Zimmerm., and Penicillium islandicum Sopp [26], serve as biological fungicides by inhibiting the germination of peanut rust urediniospores, thereby reducing the severity of the infection. These agents persist on peanut plants until the peanut rust pathogen develops, being released along with rust spores when the pustules burst [30].

(IV) Host resistance: various national and international crop breeding institutions have successfully developed rust-resistant peanut genotypes, including GPBD4 [31], Zhonghua No. 9 [32], and ICGV86699 [33]. Wild *Arachis* species generally exhibit higher levels of resistance to rust than *A. hypogaea* [34,35]. Efforts have been made to harness resistance from wild species through the development of interspecific hybrids and derivatives [36]. However, factors such as associated linkage drag, ploidy barriers, genetic isolation, and incompatibility limit the widespread use of resistance from wild species [37]. Host resistance is considered the most desirable strategy against peanut rust due to its ability to address the shortcomings of the other three approaches.

## 4. Breeding Peanuts for Rust Resistance

### 4.1. Genetics of Peanut Rust Resistance

Several previous research studies have indicated that rust resistance in peanuts is generally a recessive trait and is controlled by multiple genes [38,39,40,41,42,43]. However, the specific number and nature of the genes governing peanut rust resistance require further investigation. According to a report produced by Singh and Moss [44], rust resistance derived from diploid wild species may be governed by a partially dominant gene. Additional studies by Wynne et al. [45] and Kokalis-Burelle et al. [26] suggest that partial resistance to rust involves several minor genes, resulting in reduced infection frequency and an extended incubation period.

Moreover, the expression of rust resistance seems to be predominantly influenced by non-additive, additive × additive, and additive × dominance gene interactions, as reported by Hayman [46]. Subsequent work conducted by Ghewande [47] also revealed that rust resistance can be conditioned by additive, additive × additive, and additive × dominance gene effects. Despite these insights into the genetic regulation of rust resistance in peanuts, information regarding gene regulation or transcript upregulation in response to peanut rust is limited and requires further investigation.

Further research in this area will undoubtedly enhance our understanding of the molecular mechanisms underlying rust resistance in peanuts. This, in turn, has the potential to aid in the development of more effective breeding strategies for rust-resistant peanut varieties. Such advancements are crucial for sustainable peanut production, helping farmers combat the impact of peanut rust and ensuring the development of a more resilient and productive peanut crop.

### 4.2. Resources for Peanut Rust Resistance

The precise evaluation and effective utilization of rust-resistant germplasms are fundamental prerequisites for the development of rust-resistant peanut cultivars. Researchers have identified a substantial number of resources demonstrating immunity or high resistance to peanut rust disease; these originate from diverse sources such as wild *Arachis* species, interspecific derivatives, cultivated varieties, breeding materials, and landraces. These evaluations have been conducted in laboratory, greenhouse, and field trials, and some of these valuable resources have already been successfully integrated into breeding programs.

Rust resistance resources can be broadly classified into two main groups:

(I) Rust resistance resources in wild *Arachis* species (Table 1) [48,49,50,51]. Wild *Arachis* species act as potential sources of novel genes that can be leveraged to enhance rust resistance in cultivated peanuts (*A. hypogaea* L.). Various studies have documented the immunity or high levels of resistance of different wild species. For example, Subrahmanyam et al. [48] identified 64 accessions of wild species that were immune to peanut rust, spanning various taxonomic sections. Additionally, six accessions, including ICG 8126, ICG 8125, ICG 8137, ICG 8952, ICG 8954, and GKP 9893, exhibited high levels of resistance, while *Arachis monticola* PI263393 displayed resistance to peanut rust. Pande and Rao [49] further identified sixty-seven accessions from seventy-four wild species, showcasing varying levels of rust resistance, including one immune accession (ICG 8954) and sixty-four highly resistant accessions. Notably, certain accessions, such as ICG 8123, ICG 8138, ICG 8216, ICG 8190, and ICG 8954, were reported to have different rust resistance levels by both Subrahmanyam et al. [48] and Pande and Rao [49]. By averaging the identification results, five accessions were classified as highly resistant, while one accession (ICG 8125) was categorized as resistant.

Moreover, under laboratory conditions, additional immune and highly resistant accessions, such as VSPmSv 13774, Pa s/n, VK 12083, VSPmSv 13710, VPoJSv 10506, VSGr 6389, and VPoBi 9230, were identified [50]. Leal-Bertioli et al. [51] also reported *Arachis magna* K 30097 to be a highly rust-resistant accession of wild species. These findings collectively highlight the diverse and promising array of rust-resistant resources available within wild *Arachis* species, underscoring their potential for contributing novel genes to enhance rust resistance in cultivated peanuts.

(II) Rust-resistant resources in cultivated species (*A. hypogaea* L.; Table 2) [35,52,53,54,55,56,57,58,59,60,61,62,63,64]. Numerous studies have been dedicated to acquiring peanut germplasm resources that exhibit immunity, high levels of resistance, or resistance to rust under diverse conditions; these include field, laboratory, glasshouse, and greenhouse trials. For instance, Liang et al. [52] highlighted Yinduhuapi as being highly resistant to rust. During field trials in Vietnam, other noteworthy genotypes, such as ICG 99051, ICG 99052, ICG 99019, ICGx950084, ICGx950166 (immune), ICG 13917, and ICG 10931 (highly resistant), were identified. Similarly, Mondal et al. [53] identified immune genotypes including GBFDS 272, DTG 27, and TFDRG 5, alongside highly resistant genotypes including NCAc 343, DTG 57, DTG 60, DTG 58, and TDG 56.

Recent studies have reported multiple genotypes that exhibit high levels of resistance to rust, such as SATGR 278-18, ICGV 00064, ICG 11426, ICG 11088, ICG 4389, ICG 6022, and ICG 6993 [54,55,56,57,58]. Additionally, various genotypes have been identified as being resistant to rust, including PI 259747, PI 390593, ICGV 94114, ICGV-SM 86021, ICGV-SM 02536, ICG 02194, ICGV 01276, and ICGV 02286. Furthermore, certain genotypes, such as DTG 60, JL 776 (immune), TG 66, and G 2-52 (highly resistant), were categorized based on their rust score. According to recent reports on peanut genotypes with varying levels of rust resistance, 31 genotypes, including ICGV 00068 and TG 60, demonstrated high levels of resistance to rust. Additionally, 11 genotypes, including ICGV-SM 05570 and Kanyomwa, exhibited resistance to rust. These findings underscore the presence of promising peanut varieties with robust resistance to rust disease, presenting significant potential for integration into breeding programs aimed at enhancing peanut rust resistance.

In conclusion, the identification and effective utilization of rust-resistant germplasms are crucial for developing rust-resistant peanut cultivars. The diverse array of resources from both wild *Arachis* species and cultivated varieties, showcasing varying levels of rust resistance, hold great promise for breeding programs focused on enhancing peanut rust resistance.

(III) The breeding lines, as detailed in Table 2 [33,35,53,54,55,61,65,66,67,68,69,70,71,72,73,74,75,76,77,78,79,80,81,82], constitute a valuable resource for elevating peanut production and global food security by adopting sustainable and disease-resistant cultivation practices. In India, six remarkable breeding lines have been identified for their immunity to rust—B3-F3-36-5, B3-F3-36-6, TFDRG 1, VG 9514, ICGV99003, and ICGV99005 [35,53,65,66]. B3-F3-36-5 and B3-F3-36-6 were meticulously developed via three backcrosses with the recurrent parent, followed by selfing in the cross between ICGV 00350 and GPBD 4, employing marker-assisted backcross breeding techniques [65]. TFDRG1, which originates from a cross between TAG 24 and V 9514, is currently in its F6 generation and was achieved by crossing cv. CO 1 with *Arachis cardenasii* [53,66]. ICGV 99003 and ICGV 99005 were crafted from interspecific populations—ICGV 99003 from [*A. hypogaea//A. duranensis/A. stenosperma*] and ICGV 99005 from [*A. hypogaea//A. batizocoi*/*A. duranensis*] [34].

Furthermore, numerous breeding lines exhibiting high levels of resistance or resistance to rust have been identified. ICGV 86699 and ICGV 87157, elite peanut germplasm lines released by ICRISAT, showcase notable resistance to rust. ICGV 86699 emerged from a single-plant selection derived from CS 29, a product of repeated selections from an interspecific population of [*A. batizocoi/A. duranensis//A. hypogaea* cv. NC 2] [33,61]. On the other hand, ICGV 87157 originated from a single-plant selection in an F_3_ population of a cross between Argentine (a Spanish cultivar) and PI 259747 (a rust- and late leaf spot (LLS)-resistant genotype) [72]. In Peru, 71 breeding lines, including ICG 1697 and ICG 10025, have been identified as having substantial resistance to rust. Additionally, other countries, such as Israel and Honduras, report 15 breeding lines (e.g., ICG 4746, ICG 7885) demonstrating high resistance to rust and 18 rust-resistant breeding lines (e.g., QT0348, ICG(FDRS)30). These findings underscore the global significance of diversified breeding lines in the pursuit of resilient and productive peanut cultivation practices.

**Table 2:** Rust-resistant germplasm resources in cultivated species and breeding lines (*A. hypogaea* L.) reported globally.

Genotype	Botanical Variety/Pedigree	Source/Origin	Rust Reaction	Reference
Yinduhuapi	-	ICRISAT	Highly resistant	Liang et al. [52]
ICGV99003, ICGV99005, ICG 99051, ICG 99052, ICG 99019, ICGx950084, ICGx950166	- -	ICRISAT	Immune	Mace et al. [35]
ICG 13917	-	ICRISAT	Highly resistant	
ICG 10931, ICG 10975, ICG 11485	-	Peru	Highly resistant	
ICG 1185	-	Argentina	Highly resistant	
ICG 11312, ICG 11325, ICG 11331	-	India	Highly resistant	
ICG 12720	-	Ecuador	Highly resistant	
GBFDS 272 DTG 27 TFDRG 5 VG 9514	- TG49 × B37c TAG 24 × VG 9514 *Arachis cardenasii ×* CO 1	India	Immune	Mondal et al. [53]
NCAc 343 DTG 57 DTG 60 DTG 58 TDG 56	NC Bunch × PI 1216067 TAG 24 × GPBD 4 TG 26 × Mutant 28-2 GPBD 4 × TG 49	India	Highly resistant	
PI 259747 (Tarapoto), 97x36HO2-1B2G3-1-2-2, 99x33-1-B2G-12-2-1,99x33-1-B2G-13-1-1,99x33-1-B2G-2-2-2,99x8-1-B2G-3-1-1,96x72-HO1-9-1-1-1-1-2-1, 98x116-5-1-1-1-2-1, 97x34-HO3-1-B2G-3-1-1-1, RP-97F2-B-9-2-2-1-b3-B, BOL3-7, DP-1, PT910-2-8-11, PTBOL3-3, PTBOL3-4, PI 568164, PI 562530	-	Peru	Resistant	Power et al. [54]
PI 568164, PI 562530	-	India	Resistant	
PI 298115	-	Israel	Resistant	
PI 540472	-	China	Resistant	
PI 314817, PI 478856	-	-	Resistant	
ISATGR 278-18	*Arachis duranesis* × *Arachis batizocoi*	ICRISAT	Highly resistant	Kumari et al. [55]
PI 390593, PI 393527A, PI 476166, PI 393531, PI 393641, PI 468363, PI 476183, NCAc17090, 203/66W CG190, NCAC 17718, EC 35399, WCG 184	-	India	Resistant	Gajjar et al. [56]
ICGs 76 2925 12697	- *A. hypogaea* -	India	Resistant	Upadhyaya et al. [57]
ICG 2381, ICG 6993	*A. hypogaea*	Brazil	Resistant	
ICG 2857	*A. hypogaea*	Argentina	Resistant	
ICG 4412, ICG 7243	*A. hypogaea*	USA	Resistant	
ICG 9037	*A. hypogaea*	Tanzania	Resistant	
ICG 9842	*A. hypogaea*	Tanzania	Resistant	
ICG 9777	*A. hypogaea*	Mozambique	Resistant	
ICG 11109	*A. hypogaea*	China	Resistant	
ICG 12000	*A. hypogaea*	Mali	Resistant	
ICG 532, ICG 9961, ICG 11109, ICG 13099	*A. hypogaea*	Unknown	Resistant	
ICG 13787	*A. hypogaea*	Niger	Resistant	
ICG 14008	*A. hypogaea*	Central African Republic	Resistant	
ICGV 00064	-	ICRISAT	Highly resistant	Sudini et al. [58]
ICG 11426	-	India	Highly resistant	
ICG 11088	-	Peru	Highly resistant	
ICG 4389	-	India	Highly resistant	
ICG 6022	-	Sudan	Highly resistant	
ICG 6993	-	Brazil	Highly resistant	
ICGs 6402, 6766	-	ICRISAT	Resistant	
92R/70-4, ICGV-SM 86021, ICGV-SM 02536, ICGV 02194, ICGV 01276, ICGV 02286, ICGV 94114	-	ICRISAT	Resistant	Okori et al. [59]
DTG 60 JL 776	TG 26 × Mutant 28-2 -	India	Immune	Mondal and Badigannavar [60]
TG 66, G 2-52	-	India	Highly resistant	
ICGVs 00068, 00246, 00248, 01274, 02323, 02411, 02446, 04087, 05036, 05100, 05141, 05163, 06142, 07235, 86699, 99051, 99052 00362, 03043, 99160	*A.cardenasii* *A. hypogaea*	ICRISAT	Highly resistant	Chaudhari et al. [61]
SPS 2, SPS 8, SPS 11, SPS 20 49 M- 1-1, 49 M-16, ICG 11337	*A. villosa* *A. hypogaea* *A. cardenasii*	India	Highly resistant	
ICGV-SM 15510	ICGV 93437 × ICGV 95342	ICRISAT	Highly resistant	Daudi et al. [62]; Daudi et al. [63]
ICGV-SMs 05570, ICGV-SMs 06737, 15524, 15559, 15567, ICGV-SMs 08584, 08587, 15546, ICGV 94124, ICG 12725	- - -	ICRISAT	Resistant	
Kanyomwa	-	Tanzania	Resistant	
DTG 60 DTG 57 GFDS 272 GPBD 5	TG 26 × Mutant 28-2 TAG 24 × GPBD 4 - TAG 24 × VG 9514	India	Highly resistant	Mondal et al. [64]
B3-F3-36-5, B3-F3-36-6 TFDRG 1	- *A. vulgaris*	India	Immune	Rajarathinam et al. [65]; Badigannavar et al. [66]
ICGs NRCGs 10121, 10123, 10950, 11072, 11108, 11597, 12487, 12510, 12565, 12566, 12718, 12899, 12900, 12912, 12915, 12925, TFDRG2, JG4_81, JG4_43, JG2-3_14	- *A. vulgaris* -	India	Resistant	Chuni et al. [67]; Badigannavar et al. [66]; Yeri and Bhat [71]
ICGV 87157, ICGV99001, ICGV 86687 (CS 16–B2–B2), BC1F3-4, BC1F3-186, BC1F3-327, RBC2F5R12_13, RBC2F5R12_15, RBC2F5R12_16, RBC2F5R12_17, RBC2F5R12_18, RBC2F5R12_19, RBC2F5R12_23, RBC2F5R12_25, RBC2F5R12_29, RBC2F5R12_30, RBC2F5R12_45, RBC2F5R12_46, RBC2F5R12_78, RBC2F5R12_87, RBC2F5R12_88, RBC2F5R12_97, RBC2F5R12_138, RBC2F5R12_139, RBC2F5R12_140, RBC2F5R12_143, RBC2F5R12_103, RBC2F5R12_104, RBC2F5R12_107, RBC2F5R12_108, RBC2F5R12_114, RBC2F5R12_117, RBC2F5R12_118, RBC2F5R12_129, RBC2F5R12_130, RBC2F5R12_133	*A. fastigiata* - -	ICRISAT	Highly resistant	Nigam et al. [72]; Reddy et al. [33]; Mace et al. [35]; Deshmukh et al. [73]; Varshney et al. [74]
ICGVs 13200, 13206, 13192, 13193, 13228, 13229, ICGV 87354 ICGV 92267 AB-ICGS76-7-1, AB-ICGS76-16-1, AB-ICGS76-18-4, AB-ICGS76-26-4, AB-ICGS76-40-6, AB-DH 86-47-1, AB-DH 86-8-2, AB-DH 86-8-4, BC1F3-76, BC1F3-278, BC1F3-296	- *A. fastigiata* -	ICRISAT	Resistant	Reddy et al. [75]; Upadhyaya et al. [76]; Kumari et al. [55]; Pasupuleti et al. [77]
ICGs 1697, 7296, 7630, 7886, 7890, 7893, 7895, 7896, 10014, 10020, 10021, 10022, 10025, 10030, 10031, 10032, 10034, 10037, 10039, 10042, 10047, 10048, 10049, 10051, 10052, 10053, 10054, 10057, 10058, 10059, 10060, 10061, 10062, 10063, 10064, 10065, 10067, 10068, 10069, 10073, 10074, 10888, 10915, 10918, 10925, 10927, 10928, 10932, 10933, 10935, 10937, 10939, 10940, 10943, 10945, 10954, 10962, 10963, 10964, 10966, 10969, 10974, 10978, 11073, 11080, 11088, 11108, 11182, 11183, 11285 ICGs 7891	*A. fastigiata* *A. hypogaea*	Peru	Highly resistant	Subrahmanyam et al. [78]
ICG 4746, ICG 7883, ICG 7884, ICG 9185	*A. hypogaea* *A. fastigiata*	Israel	Highly resistant	
ICG 6330	*A. hypogaea*	Zimbabwe	Highly resistant	
ICG 6340 ICG 7899, ICG 7900	*A. fastigiata* *A. hypogaea*	Honduras	Highly resistant	
ICG 7621	*A. hypogaea*	USA	Highly resistant	
ICG 7885	*A. fastigiata*	Honduras	Highly resistant	
ICG 7897	*A. fastigiata*	Venezuela	Highly resistant	
ICG 8044	*A. fastigiata*	South Africa	Highly resistant	
ICG 10884	*A. hypogaea*	Bolivia	Highly resistant	
QT0348, QT0368, QT0400, QT0402, QT0419, QT0458, QT0463, QT0485	-	China	Resistant	Cheng et al. [79]
Tifrust-5 (GP 22), Tifrust-6 (GP 23), Tifrust-7 (GP 24), Tifrust-8 (GP 25), Tifrust-9 (GP 26), Tifrust-10 (GP 27), Tifrust-11 (GP 28), Tifrust-12 (GP 29), Tifrust-13 (GP 30)	-	USA; ICRISAT	Resistant	Hammons et al. [80,81]
ICG(FDRS)30	-	USA; ICRISAT; Israel	Resistant	Reddy et al. [82]

Note: “-”: details not available.

### 4.3. Breeding for Rust Resistance

Breeding rust-resistant peanuts constitutes a pivotal objective in peanut breeding programs to ensure the enduring sustainability and growth of global peanut production. In recent decades, substantial strides have been made in developing numerous rust-resistant cultivars and breeding lines of *A. hypogaea* L., leveraging both traditional breeding methods and modern molecular breeding technologies.

The development and release of rust-resistant peanut varieties represent significant achievements in peanut breeding programs (Table 3) [31,32,53,54,56,62,63,67,83,84,85,86,87,88,89,90,91,92,93,94,95,96,97,98,99,100,101,102,103,104,105,106,107,108,109,110,111,112,113,114,115,116,117,118,119], contributing substantially to enhanced crop productivity and mitigating yield losses attributed to rust disease. In India, three noteworthy peanut varieties—GPBD 4, Mutant 28-2, and ICGV 86590—have attracted considerable attention for their robust rust resistance. GPBD 4, a promising early-maturing variety released in Karnataka, showcases high levels of resistance to rust. This achievement resulted from a cross between KRG 1 (a susceptible cultivar) and ICGV 86855 (a resistant genotype), which is an interspecific derivative of *A. hypogaea* × *A. cardenasii* [36]. Mutant 28-2, an improved high-yielding Spanish peanut variety released in Karnataka, also boasts high levels of rust resistance. It was developed via the ethyl methane sulfonate (0.5%)-induced mutagenesis of VL 1, itself a mutant derivative of Dharwad Early Runner [83]. ICGV 86590, a rust-resistant peanut variety bred at ICRISAT, resulted from a cross between X 14-4-B-19-B (a Spanish breeding line) and PI 259747 (a rust- and late leaf spot (LLS)-resistant Valencia germplasm line) [85].

In the USA, several rust-resistant peanut varieties have been released for peanut production, including Southern Runner [84], C-99R [86], Florida MDR 98 [87], Hull [88], and York [89]. In China, peanut varieties with high levels of resistance to rust, such as Guihua 23 and yuanza 9102, were bred through distant hybridization. This involved using peanut diploid wild species *A.chacoense* with high rust resistance as the male parent and Baisha 1016 as the female parent [94,95]. Yueyou 114 [100] and Fuhua 6 [103], have been released for peanut production, achieving notable resistance outcomes. Similarly, rust-resistant varieties including Yueyou 79 [52], Guihua 21 [117], and Zhonghua No. 12 [32] have also been developed. Moreover, rust-resistant peanut varieties have been released in other countries. In the Philippines, NSIC Pn 12 [119] and in Tanzania Narinut 15 [62] and Narinut [63] have been released to combat rust disease. This comprehensive global effort underscores the widespread commitment to addressing the challenges posed by rust, ensuring a resilient and productive future for peanut cultivation.

## 5. Application of Molecular Marker Technology and the Mining of Rust Resistance Gene

Traditionally, the process of breeding peanuts for rust resistance has been characterized by the use of expensive, time-consuming, and sometimes inefficient methods, including artificial inoculation and a reliance on natural occurrences when assessing resistance levels. However, a transformative shift has occurred with the advent of molecular peanut breeding, offering a more efficient and precise approach that can substantially shorten the breeding cycle and enhance overall selection efficiency. Molecular marker technology, a cornerstone of molecular breeding, has proven instrumental in peanut breeding by facilitating the tagging of genes and the selection of rust-resistant genotypes. Various types of molecular markers, such as Amplified Fragment Length Polymorphism (AFLP) markers, Random Amplified Polymorphic DNA (RAPD) markers, Simple Sequence Repeat (SSR, microsatellites) markers, and Single Nucleotide Polymorphism (SNP) markers, have been effectively employed in this revolutionary process. For example, in a study involving the cross Yuanza 9102 × ICGV86699, the researchers identified two AFLP markers, M3L3-46 and M8L8-645, which are linked to rust resistance, at genetic distances of 10.9 cM and 7.86 cM, respectively [120]. In another investigation using an F_2_ mapping population from VG 9514 × TAG 24, two RAPD markers, J71350 and J71300, were identified. J71350 was linked to the rust resistance gene at a distance of 18.5 cM, while J71300 was completely linked to rust resistance [121]. Additionally, certain SSR alleles in crosses ICGV99005 × TMV2 and ICGV99003 × TMV2 were found to be associated with rust resistance [122]. Other studies identified candidate SSR loci for mapping rust resistance using an analysis of molecular variance and Kruskal–Wallis one-way ANOVA [35,123]. In a separate investigation involving a population of 164 recombinant inbred lines (RILs) derived from VG 9514 (a rust-resistant genotype) × TAG 24 (a susceptible genotype), the researchers assessed rust resistance in the field and generated a genetic linkage map with 24 linkage groups (LGs) using 109 SSR markers. Notably, SSR markers gi56931710 and pPGPseq4A05 were identified as flanking the rust resistance gene at map distances of 4.3 cM and 4.7 cM in the linkage group (LG) 2, respectively [53]. In conclusion, peanut molecular breeding that leverages molecular markers has demonstrated significant potential in enhancing the efficiency and precision of rust resistance selection; this constitutes a notable advantage over traditional breeding methods. The integration of these technologies is crucial for ensuring sustainable and disease-resistant peanut production on a global scale.

With the rapid evolution of molecular marker technology, numerous quantitative trait loci (QTLs) linked to peanut rust resistance have emerged, driven by high-density linkage maps and detailed phenotypic data (Table 4) [60,77,78,79,80,81,82,120,121,122,123,124,125,126,127]. In one notable study, 12 QTLs governing rust resistance were pinpointed using composite interval mapping (CIM) within a cohort of 268 recombinant inbred lines (RILs) derived from the cross TAG 24 × GPBD 4. These QTLs were meticulously assessed using 67 polymorphic SSR markers, leading to a key discovery: the identification of the candidate SSR marker IPAHM 103, which is firmly linked to a major QTL (QTLrust01) correlated with rust resistance. This marker explained a phenotypic variation ranging from 6.9% to 55.2% [123]. Another investigation, employing two distinct RIL populations (TAG 24 × GPBD 4 - RIL-4 and TG 26 × GPBD 4 - RIL-5), revealed a total of 15 QTLs associated with rust resistance. Among these, RIL-4 exhibited three significant QTLs (QTLR4-Rust01, QTLR4-Rust02, and QTLR4-Rust03), while RIL-5 showcased four major QTLs (QTLR5-Rust01, QTLR5-Rust02, QTLR5-Rust03, and QTLR5-Rust04). These QTLs exhibited a noteworthy spectrum of phenotypic variations, spanning from 10.68% to an impressive 82.96%. Additionally, five SSR markers (IPAHM103, GM2009, GM1536, GM2301, and GM2079) exhibited significant associations with the major QTLs, collectively elucidating a substantial phenotypic variation of 82.96% [124]. Moreover, within a specific genomic region on LG AhXV, flanked by GM2009 and GM1954 markers, three robust QTLs for rust resistance were localized, demonstrating an explained phenotypic variance (PVE) of up to 82.96% [128]. In a separate study, involving an F_6 population derived from a cross between *A. ipaënsis* K30076 and *A. magna* K30097, the researchers identified thirteen QTLs governing rust resistance, with two being classified as major. One of these major QTLs (PVE 5.8% to 59.3%) was situated between 35.1 and 42.9 cM on LG B08, with the closest microsatellite marker of Ah-280. The second major QTL (PVE 13.2% to 34.8%) was mapped between microsatellite markers AHGS1350 and AHGS2541, spanning positions 25.4 to 33.1 cM on the same LG B08. Additionally, competitive allele-specific PCR markers were crafted and validated on a tetraploid background for these QTLs [51]. These findings showcase the prowess of molecular marker technology in not only identifying but also characterizing QTLs that are bound up with rust resistance in peanuts. Such insights hold a great deal of promise for breeding programs dedicated to cultivating rust-resistant peanut varieties.

Numerous candidate genes associated with peanut rust resistance have been meticulously identified, as outlined in Table 5 [60,127,128,129,130]. One pivotal quantitative trait locus (QTL) of rust, referred to as Rust-QTL, which is responsible for reducing the lesion number, size, and sporulation of rust, was discerned in *Arachis magna* K30097. This locus, situated on LG B8, is flanked by the closest linked marker, Ah280, which maps near an NB-LRR-encoding gene known as Araip.RV63R [129]. In a separate study, an enhanced genetic map featuring 20 linkage groups (LGs), incorporating 139 new SSR and transposable element markers, was deployed for QTL mapping for rust resistance in a recombinant inbred line (RIL) population derived from TAG 24 (a susceptible variety) and GPBD 4 (a resistant variety) [125]. The results of the analysis revealed five QTLs associated with rust (with the explained phenotypic variance, PVE, ranging from 44.5% to 53.7%) on AhXV (B03 LG of the B genome), flanked by GM2009-IPAHM103. Additionally, a QTL linked to rust (PVE 49.3-52.3%) was identified on AhV (A05 LG of the A genome), flanked by GM1989-AhTE0839 [125]. Diligent efforts were undertaken to construct a high-density genetic map of TAG 24 × GPBD 4, encompassing 29 LGs with 453 loci (including 171 SNPs, 89 transposons, and 193 SSRs) covering a span of 1510.1 cM. The subsequent QTL analysis identified nine QTL candidates for rust resistance; they were concentrated in a genomic region of 2.7 Mb (131.9–134.6 Mb on A03). Within these regions, six genes with deleterious mutations (Aradu.C88Z1, Aradu.Z87JB, Aradu.1WV86, Aradu.RW91L, Aradu.NG5IQ, and Aradu.YL3ZN) emerged as potential candidates for rust resistance. This determination was made through ddRAD-Seq analysis, linkage map construction, QTL analysis, sequencing, and whole-genome resequencing analysis [127]. To further define the rust resistance gene, an RIL population resulting from a cross between VG 9514 and TAG 24 was subjected to phenotyping and subsequent QTL analysis. The results revealed a consensus rust QTL (Rust_QTL) flanked by two SSR markers (FRS72 and SSR_GO340445) within a 1.25 cM map interval on the A03 chromosome in cultivated peanuts. This region encompasses an R-gene (Aradu.Z87JB; TIR–NB–LRR) and four PR-genes (Aradu.RKA6 M, Aradu.T44NR, Aradu.1WV86, and Aradu.VG51Q) associated with rust resistance [60]. Additionally, a conserved Tir-NBS-LRR gene (AH13G54010.1), residing in a common genomic region colocalized with resistance to rust and LLS, appeared to translocate from Chr03 to Chr13 after tetraploidization; thus, it represents a potential candidate for resistance to rust and LLS [130]. In a subsequent analysis involving 328 F_2_ individuals derived from a cross between two Indian varieties (GJG17 × GPBD4), a major QTL for rust resistance (RustQTL; PVE 70.52%) was validated. This QTL was found to be flanked by markers SSR_GO340445 and FRS72, situated on the A03 chromosome. Notably, in addition to the five genes identified by Mondal and Badigannavar [60], a PR gene (Aradu.RW91L) encoding lipase/lipooxygenase was validated in the RustQTL region, being identified as contributing significantly to rust resistance. Furthermore, seven novel EST-SSRs were validated in 177 peanut genotypes [128]. These comprehensive findings shed light on the intricate genetic landscape governing peanut rust resistance, offering valuable insights for future breeding programs aimed at developing resilient peanut cultivars.

The isolation of the dominant rust resistance gene, VG 9514-Rgene (GenBank accession number MK791522), was successfully achieved using map-based cloning, employing VG 9514, TAG 24, and 164 recombinant inbred lines (RILs) derived from the cross of VG 9514 × TAG 24 [64]. A BLASTn search identified six genes homologous to VG 9514-Rgene, namely Aradu.Z87JB (Aradu.A03; in *A. duranensis*), Araip.0R3VU (Araip.B03; in *A. ipaensis*), Arahy.GFGJ54 (Arahy13; in *A. hypogaea*), Arahy.T6DCA5 (Arahy03; in *A. hypogaea*), Arahy.R8KUIR (Arahy03; in *A. hypogaea*), and Arahy.ZZ0VZ9 (Arahy03; in *A. hypogaea*) [64]. The genomic location of VG 9514-Rgene was predicted to be at 142,544,745.0-142,549,184 bp on chromosome arahy03, coinciding with the location of Arahy.T6DCA5. Further analysis of VG 9514-Rgene revealed its structural composition, encoding a typical consensus three-dimensional folding of the TIR-NBS-LRR protein. This protein class is well-known for its role as a resistance (R) protein and is integral in plant defenses against pathogens. Using sequence analysis, three non-synonymous mutations (E268Q in the hhGRExE motif, Y309F in the RNBS-A motif, and I579T in the MHD motif of the NB-ARC domain) were identified in the susceptible version of the R-protein. These mutations were mapped and found to be associated with the loss of the rust resistance function in the susceptible version of the gene [53]. The elucidation of the molecular structure and mutational landscape of VG 9514-Rgene provides crucial insights into the mechanisms underlying rust resistance in peanuts. This information holds significant potential for the development of rust-resistant cultivars through both traditional breeding methods and advanced biotechnological approaches. By understanding the genetic basis of resistance, breeders and biotechnologists can make informed decisions to enhance the efficiency and precision of crop improvement programs, ultimately contributing to sustainable peanut cultivation practices.

## 6. Outlook

Sustainable peanut production is imperative for global food security given their high nutritional value and abundant seed oil content. However, peanut productivity faces significant challenges, notably peanut rust and abiotic stresses that impede production worldwide. The various proposed control strategies include cultural, chemical, and biological approaches, but each has inherent limitations. Large-scale biological control methods encounter challenges including constraints related to biocontrol fungi, complex operations, unstable control effects, and high costs. Consequently, peanut research has shifted its focus toward breeding immune or highly resistant varieties as the most environmentally friendly, economical, and effective strategy for rust control.

Like other crops, peanuts can achieve enduring resistance if rust immunity or resistance genes are introduced from elite sources into genotypes adapted to diverse production regions. Conventional breeding methods have successfully integrated resistance traits, although it is crucial to note that only specific wild *Arachis* species in *Arachis* areas possess immunity or high levels of rust resistance. Interspecific hybridization using these wild relatives can transfer rust resistance to cultivated peanut varieties. By harnessing the genetic diversity in wild relatives, peanut breeders can develop rust-resistant varieties suited to specific agro-climatic conditions, contributing to sustainable and enhanced global peanut production. The ongoing efforts to develop rust-resistant varieties play a crucial role in ensuring food security and improving the livelihoods of peanut farmers worldwide.

Advancements in molecular breeding techniques, including molecular markers, transgene technology, and molecular design, are leading peanut breeding to undergo a transition from traditional “experience breeding” to efficient, precise, and targeted “molecular design breeding.” The rapid progress in sequencing technology has facilitated genome sequences for various *Arachis* species. The whole-genome sequencing of diploid peanut ancestors (*A. duranensis* and *A. ipaensis*) and allotetraploid peanuts (*A. hypogaea* L.) has provided valuable genomic information. This has accelerated the identification and isolation of rust-resistant genes, enhancing the discovery of more rust-resistant genes.

Precision-designed breeding enables the direct selection and efficient stacking of rust-resistant genes, representing the future of breeding peanuts for rust resistance. This approach offers advantages such as a shortened breeding period, the effective resolution of issues related to a narrow genetic basis and limited adaptability, and improved efficiency in achieving rust-resistant varieties. With the integration of molecular design techniques, peanut breeding programs are positioned to achieve enhanced rust resistance more rapidly and effectively, thereby contributing to sustainable peanut production and global food security.

## 7. Conclusions

Understanding and managing peanut rust disease is crucial for securing a prosperous future for peanut production and meeting the escalating demand for this nutritious and versatile crop. This review offers an extensive elucidation of peanut rust disease and the breeding of rust-resistant cultivars, describing the disease’s characteristics and detrimental symptoms, its epidemiology, and diverse control strategies. Additionally, the review emphasizes the utilization of molecular marker technology and the identification of rust-resistant genes. This comprehensive compilation of information serves as a valuable resource for breeders and biotechnologists, empowering them to collaboratively develop immune or resistant peanut varieties, ensuring the sustainability and vitality of the peanut industry.

## Figures and Tables

**Table 1:** Rust-resistant resources in wild *Arachis* species reported globally.

Species	Genotype	Source/Origin	Rust Reaction	Reference
*A. batizocoi*	ICG 8124	ICRISAT	Immune	Subrahmanyam et al. [48]
*A.correntina* ^a^	ICGs 4984, 8134, 8132, 8140			
*A.chacoense* ^a^	ICG 4983			
*A.villosa*	ICG 8144			
*Arachis* sp. ^b^	ICG 8918			
*Arachis* sp.	ICGs 8193, 8127, 8128, 8145, 8148, 8152, 8154, 8155, 8156, 8158, 8929, 8159, 8160, 8161, 8162, 8165, 8166, 8933, 8167, 8168, 8170, 8171, 8941, 4984, 8937			
*A.apressipila* ^a^	ICG 8129			
*A.paraguariensis*	ICG 8130			
*A.pusilla*	ICG 8131			
*A.villosulicarpa*	ICG 8142			
*A.hagenbeckii*	ICGs 8922, 8146			
*A.glabrata*	ICGs 8149, 8150, 8151, 8153, 8935, 8936, 8902			
-	ICGs 8172, 8916			
*A. stenosperma* ^a^	ICGs 8126, 8137, 8952, 8954, GKP9893	ICRISAT	Highly resistant	Subrahmanyam et al. [48]
*A.glabrata* *A.villosulicarpa*	PIs 118457, 231318, 262287, 262141, 262801, PI 336985	-	Immune	
*A.monticola*	PI 263393	-	Immune	
*A. duranensis*	ICGs 8123, 8138, 8139, 8195, 8196, 8199, 8200, 8201, 8202, 8204, 8205, 8957, 11550, 11552, 11553, 11554, 11555, 12162, 13161, 13174, 13175, 13176, 13183, 13184, 13185, 13186, 13189, 13190, 13191, 13192, 13194, 13195, 13197, 13199, 13200, 13201, 13202, 13203, 13205, 13206, 13207, 13217, 13242, 15179	ICRISAT	Highly resistant	Pande and Rao [49]
*A. hoehnei*	8190, 14867			
*A. kretschmeri*	8191			
*A. cardenasii*	8216, 11558, 11566			
*A. batizogaea*	8901, 13208			
*A. stenosperma*	8906, 13173, 13233, 14868, 14872			
*A. kempff-Mercadoi*	8959			
*A. magna*	8960			
*A. valida*	11548			
*A. benensis*	11551			
*A. chiquitana*	11560			
*A. decora*	14939			
*A. kuhlmannii*	15144			
*A. stenosperma* *A. villosa*	ICG 8125 ICG 8144	ICRISAT	Resistant	Pande and Rao [49]
*A. aff. diogoi* *A. helodes* *A. simpsonii* *A. kuhlmannii* *A. gregoryi* *A. kuhlmannii*	VSPmSv 13774 Pa s/n, VK 12083 VSPmSv 13710 VPoJSv 10506 VSGr 6389 VPoBi 9230	Brazil	Immune	Fávero et al. [50]
*A. cardenasii*	GKP 10017, 1	Brazil	Highly resistant	
*A. helodes*	CoSzSv 6862, VPoJSv 10470, VMPzW 13985, KG 30006, VPoBi 9094, VSGr 6325, VPoBi 9146			
*A. linearifolia*	VPoBi 9401			
*A. stenosperma*	VGaRoSv 12488, HLK 408, Lm 5, VSStGdW 7762, Jt 2, Lm 3, SvW 3755, VKSSv 9010, VMiSv 10229, VSPmSv 13832, WPz 422, Lm 1, VSSv 7382, VSv 10309, VSPmSv 13670, VKSSv 9017, WPz 421, SvPzSz 3042, VGaSv 12646, VSPmWiSv 13262, VSPmSv 13693, VSPmW 13844, VSSv 13258			
*A. diogoi*	GK 10602			
*A. duranensis*	VNvEv 14167, K 7988			
*A. kempff-mercadoi*	V 13250			
*A. kuhlmannii*	VRGeSv 7639, VPoBi 9235, VSGr 6344, VSGr 6352, VSGr 6380, VKSSv 8916a, VPoBi 9470, VPoBi 9479, VSW 9912, VSPmSv 13721, VSGr 6351, VKSSv 8979, VPoBi 9394, VSGr 6413, VPoBi 9375, VPoBi 9214, VPoBi 9243			
*A. microsperma*	VRGeSv 13545, VMPzW 14042			
*A. magna*	KGSSc 30097, VSPmSv 13751, VSPmSv 13765, VSPmSv 13748			
*A. villosa*	VGoMrOv 12812			
*A. batizocoi*	K 9484 mut			
*A. cruziana*	WiSVg 1302			
*A. simpsonii*	VSPmSv 13716, VSPmSv 13728, VSPmSv 13745			
*A. schininii*	VSW 9923			
*A. valida*	VPzRcSgSv 13514			
*A. magna*	K 30097	Brazil	Highly resistant	Leal-bertioli et al. [51]

“-”: details not available; “a”: *Nomen nudum*; “b”: A hybrid between *A. correntina* and *A. villosa*.

**Table 3:** Peanut cultivars resistant to *P. arachidis* Speg.

Genotype	Source/Origin	Rust Reaction	Reference
GPBD 4, Mutant 28-2	India	Highly resistant	Gowda et al. [31,83]; Mondal et al. [53]
Ah 6, ALR 1, ALR 2, ALR 3, BSR 1, Chitala White, CSMG 84-1, Girnar 1, ICG FDRS 10, ICGS 76, JGN 3, KRG 1, R2001-2, R2001-3, VG0401, VG0411, VG0430, VG0437, VG0438, VG09405, VG09406, VRIGn 5	India	Resistant	Chuni et al. [67]; Gajjar et al. [56]
ICGV 86590; ICGV 86855	ICRISAT	Resistant	Gowda et al. [31,83]; Power et al. [54]
Southern Runner, Florida MDR 98, C-99R, Hull, York	USA	Resistant	Gorbet et al. [85]; Gorbet and Shokes [86,87]; Gorbet [88]; Gorbet and Tillman [89]
Fuhua 6, Guihua 23, Kainongheihuasheng, Quanhua 7, Shangyan 9658, Shanyou 27, Shanyou 65, Shanyou 71, Shanyou 162, Shanyou 212, Shanyou 523, Yuanza 9102, Yuanza 9847, Yueyou 7, Yueyou 9 *, Yueyou 14 *, Yueyou 93, Yueyou 114, Yueyou 223, Yuhua 9840 *, Zhanyou 30, Zhanyou 62, Zhanyou 75, Zhongkaihua No. 1	China	Highly resistant	Zhen and Chen [90]; Liang et al. [91]; Zheng et al. [92]; Zheng and Chen [93]; Zhong et al. [94]; Wu et al. [95]; Chen et al. [96]; Feng et al. [97]; Zheng et al. [98]; Fang et al. [99]; Li et al. [100]; Wu et al. [101]; Chen et al. [102]; Tang et al. [103]; Chen et al. [104]; Wang et al. [105]; Li et al. [106]; Li et al. [107]; Su and Huang [108]; Chen et al. [109]; Xu et al. [110]
Yueyou 5, Yueyou 79, Zhonghua No. 4, Zhonghua No. 9, Zhonghua No. 12, Yuhua No. 15, Shangyan 9938, Huayu 18, Yueyou 92, Guihua 21, Quanhua 327, Tianfu 22	China	Resistant	Liang et al. [52]; Tang et al. [111]; Liao and Lei [32]; Liao and Lei [112]; Wu et al. [113]; Su et al. [114]; Mao et al. [115]; Ye [116]; Zhou [117]; Chen et al. [118]
NSIC Pn 12	Philippines	Resistant	Sugui [119]
Narinut 15, Narinut	Tanzania	Resistant	Daudi et al. [62]; Daudi et al. [63]

“*”: variety has been approved but not reported in a journal.

**Table 4:** Quantitative trait loci (QTL) conferring resistance to groundnut rust.

Major QTL	Linkage Group/Chromosome	Marker Interval	Position (cM)	LOD Value	Phenotypic Variance Explained (PVE%)	Population	Reference
QTL01 *	AhXV (B03)	AhTE0498–GM2009	0.0–17.8	21.4–30.9	62.7–70.4	TAG 24 × GPBD 4	Kolekar et al. [124]
QTL02 *	AhXV (B03)	GM2079–AhTE0928	34.0–40.3	14.3–56.0	19.4–64.6	TAG 24 × GPBD 4	
QTL03 *	AhXV (B03)	GM2009–IPAHM103	17.8–26.8	31.3–54.5	30.0–53.7	TAG 24 × GPBD 4	
QTL04 *	AhXV (B03)	IPAHM103–GM2301	26.8–29.3	40.9–42.3	34.7–35.9	TAG 24 × GPBD 4	
QTL05 *	AhXV (B03)	GM2301–GM1536	29.3–31.7	35.3	34	TAG 24 × GPBD 4	
QTL06 *	AhV (A05)	GM1989–AhTE0839	65.8–80.5	2.3	10.2	TAG 24 × GPBD 4	
*qRust80D_06*	A03	GMRQ517–Seq2B10	31.6	36.1	83.6	TAG 24 × GPBD 4	Pandey et al. [125]
*qRust90D_06*	A03	GMRQ517–Seq2B10	30.6	24.1	75.4	TAG 24 × GPBD 4	
*qRust80D_07*	A03	GMRQ517–Seq2B10	31.6	49.9	65.4	TAG 24 × GPBD 4	
*qRust90D_07*	A03	GMRQ517–Seq2B10	31.6	47.2	73.1	TAG 24 × GPBD 4	
*qRust80D_08*	A03	GMRQ843–Seq2B10	31.6	35.2	69.7	TAG 24 × GPBD 4	
*qRust90D_08*	A03	GMRQ517–Seq2B10	31.6	49.2	63.7	TAG 24 × GPBD 4	
*qRust80D_09*	A03	GMRQ517–Seq2B10	31.6	16	48.9	TAG 24 × GPBD 4	
*qRust90D_09*	A03	GMRQ517–Seq2B10	31.6	14.6	42.7	TAG 24 × GPBD 4	
QTL-1 *	AhXV (A03)	GM2009–GM2079	7.77–13.89	4.05–4.96	7.59–11.77	TAG 24 × GPBD 4	Shirasawa et al. [126]
QTL-2 *	AhXV (A03)	IPAHM103–Aradu.A03_AhTE0498	19.49–26.66	6.78–26.14	14.86–35.72	TAG 24 × GPBD 4	
QTL-3 *	AhXV (A03)	GM1954–Aradu.A03_134423742	28.74–30.99	5.2	8.33	TAG 24 × GPBD 4	
QTL-4 *	AhXV (A03)	Aradu.A03_133094130–Aradu.A03_132351805	33.65–33.76	5.61	8.71	TAG 24 × GPBD 4	
QTL-5 *	AhXV (A03)	Aradu.A03_132351826–Aradu.A03_132071793	33.80–33.91	2.91	3.94	TAG 24 × GPBD 4	
QTL-6 *	AhXV (A03)	Aradu.A03_132071793–Aradu.A03_132512710	33.91–33.96	7.34–15.94	8.99–22.06	TAG 24 × GPBD 4	
QTL-7 *	AhXV (A03)	Aradu.A03_133038951–Aradu.A03_133449043	34.06–34.09	6.22	6.81	TAG 24 × GPBD 4	
QTL-8 *	AhXV (A03)	Aradu.A03_133398903–Aradu.A03_132771430	34.42–34.50	9.45	14	TAG 24 × GPBD 4	
QTL-9 *	AhXV (A03)	Aradu.A03_131792747–Aradu.A03_133997678	35.69–35.86	8.25	12.42	TAG 24 × GPBD 4	
Rust_QTL	A03	FRS72–SSR_GO340445	109.8-111.0	68.0–88.4	68.0–95.3	VG 9514 × TAG 24	Mondal and Badigannavar [60]
Rust_QTL_	A03	FRS72–SSR_GO340445	133	87.81	70.52	GJG17× GPBD4	Ahmad et al. [127]

“*”: Major QTLs are not named by the researchers but by the authors of this review in order to distinguish them from others.

**Table 5:** Candidate genes for rust resistance.

CDS_ID/ Gene ID	Chromosome	Gene Position Start	Gene Position End	Annotation	Amino Acid Sequence Mutation	Reference
*Araip.RV63R*	Araip.B08	127081618	127106493	Disease resistance protein (TIR-NBS-LRR class)	-	Bertioli et al. [129]
*Aradu.C88Z1*	Aradu.A03	133033579	133038386	Seed linoleate 9S-lipoxygenase	His307Arg, Gly49Arg, and Leu34Ser	Shirasawa et al. [127]
*Aradu.YL3ZN*	Aradu.A03	134333421	134335845	Receptor-like kinase 1	Arg47Ser	Shirasawa et al. [127]
*AH13G54010.1*	Chr13(B03)	143852400	143858431	Disease resistance protein (TIR-NBS-LRR class)	-	Zhuang et al. [130]
*Aradu.RW91L*	Aradu.A03	133933250	133935646	Lipase/lipooxygenase, PLAT/LH2 family protein	Glu344Ala	Shirasawa et al. [127]; Ahmad et al. [128]
*Aradu.T44NR*	Aradu.A03	133870461	133871403	Glucan endo-1,3-beta-glucosidase-like protein 3-like [Glycine max]	-	Mondal and Badigannavar [60]; Ahmad et al. [128]
*Aradu.RKA6 M*	Aradu.A03	133868497	133869566	Glucan endo-1,3 β glucosidase like protein-3 like [Glycine max]	-	Mondal and Badigannavar [60]; Ahmad et al. [128]
*Aradu.1WV86*	Aradu.A03	133878019	133879319	Glucan endo-1,3-beta-glucosidase-like protein 2-like [Glycine max]	Cys8Tyr	Shirasawa et al. [127]; Mondal and Badigannavar [60]; Ahmad et al. [128]
*Aradu.NG5IQ*	Aradu.A03	133995919	133999850	Glucan endo-1,3-beta-glucosidase 4-like [Glycine max]	Lys127Glu, Pro116Leu, Ser72Cys, and Gly69Cys	Shirasawa et al. [127]; Mondal and Badigannavar [60]; Ahmad et al. [128]
*Aradu.Z87JB*	Aradu.A03	133776796	133780539	Disease resistance protein (TIR-NBS-LRR class), putative	Ile27Val	Bertioli et al. [129]; Shirasawa et al. [127]; Mondal and Badigannavar [60]; Ahmad et al. [128]

“-”: details not available.

## Data Availability

Not applicable.

## References

[1] Food Agriculture Organization of the United Nations (2021). FAOSTAT. Food and Agriculture Organization of the United Nations: Rome, Italy. http://faostat.fao.org.

[2] Leppik E.E. (1971). Assumed gene centers of peanuts and soybeans. Econ. Bot..

[3] Hennen J.F., Figucredo M.B., Riberio I.J.A., Soave J. (1976). The occurrence of teliospores of *Puccinia arachidis* (Uredinales) on *Arachis hypogaea* in Sao Paulo State, Brazil. Summa Phytopathol..

[4] Bromfield K.R. (1971). Peanut rust: A review of literature. J. Am. Peanut Res. Educ. Assoc..

[5] Mondal S., Badigannavar A.M. (2015). Peanut rust (*Puccinia arachidis* Speg.) disease: Its background and recent accomplishments towards disease resistance breeding. Protoplasma.

[6] Daudi H., Shimelis H., Mwadzingeni L., Laing M., Okori P. (2019). Breeding peanut for rust resistance: A review. Legume Res..

[7] Subrahmanyam P., McDonald D. (1987). Peanut rust disease: Epidemiology and control. Peanut Rust Disease, Proceedings of the Discussion Group Meeting, Patancheru, India, 24–28 September 1984.

[8] Kirk P.M., Cannon P.F., Minter D.W., Stalpers J.A. (2008). Dictionary of the Fungi.

[9] Aime M.C., Toome M., McLaughlin D.J., McLaughlin D., Spatafora J.W. (2014). The Pucciniomycotina. The Mycota VII Part A. Systematics and Evolution.

[10] Tashildar C.B., Avider S.S., Chattannavar S.N., Palakshappa M.G., Kenchanagaudar P.V., Fakruddin B. (2012). Morphological and isozyme variations in *Puccinia arachidis* Speg. causing rust of peanut. Karnataka J. Agric. Sci..

[11] Rodrigues A.A.C., Silva G.S., Moraes F.H.R., Silva C.L.P. (2006). *Arachis repens*: Novo Hospedeiro de *Puccinia arachidis*. Fitopatol. Bras..

[12] Das S., Raj S.K. (1999). Temporal and spatial epidemic development of peanut rust (*Puccinia arachidis* Speg.) as a function of altered date of sowing. Trop. Agric..

[13] Foudin A.S., Macko V. (1974). Identification of the self inhibitor and some germination characteristics of peanut rust uredospores. Phytopathology.

[14] Mondal S., Hande P., Badigannavar A.M. (2014). Identification of transposable element markers for a rust (*Puccinia arachidis* Speg.) resistance gene in cultivated peanut. J. Phytopathol..

[15] Cook M. (1980). Host-parasite relations in uredial infections of peanut by *Puccinia arachidis*. Phytopathology.

[16] Leal-Bertioli S.C.M., Farias M.P., Silva P.T., Guimarães P.M., Brasileiro A.C.M., Bertioli D.J., Araujo A.C.G. (2010). Ultrastructure of the initial interaction of *Puccinia arachidis* and *Cercosporidium personatum* with leaves of *Arachis hypogaea* and *Arachis stenosperma*. J. Phytopathol..

[17] Leal-Bertioli S.C.M., José A.C.V., Alves-Freitas D.M., Moretzsohn M.C., Guimarães P.M., Nielen S., Vidigal B.S., Pereira R.W., Pike J., Fávero A.P. (2009). Identification of candidate genome regions controlling disease resistance in *Arachis*. BMC Plant Biol..

[18] Sunkad G., Kulkarni S. (2007). Studies on perpetuation and carry over of peanut rust (*Puccinia arachidis* Speg.) in northern Karnataka. Karnataka J. Agric. Sci..

[19] Park R.F., Wellings C.R. (2012). Somatic hybridization in the uredinales. Ann. Rev. Phytopathol..

[20] Peregrene W.T.J. (1971). Peanut rust *(Puccinia arachidis*) in Brunei. Pest Artic. News Summ..

[21] Subrahmanyam P., McDonald D. (1982). Peanut rust-its survival and carry over in India. Proc. Indian Acad. Sci..

[22] Rao A.S., McDonald D., Reddy K.R. (1997). Effect of temperature on rust and late leaf spot disease development in peanut. J Oilseed Res..

[23] Pivonia S., Yang X. (2006). Relating epidemic progress from a general disease model to seasonal appearance time of rusts in the United States: Implications for soybean rust. Phytopathology.

[24] Savary S., Janeau J.L. (1986). Rain-induced dispersal in *Puccinia arachidis*, studied by means of a rainfall simulator. Neth. J. Plant Pathol..

[25] Bulbule S., Mayee C. (1997). Influence of weather parameters on the deposition pattern of urediniospores of peanut rust. Indian J. Mycol. Pl. Path..

[26] Kokalis-Burelle N., Porter D.M., Rodriguez-Kabana R., Smith D., Subrahmanyam P. (1997). Compendium of peanut diseases. Am. Phytopathol..

[27] NARI (2001). Annual Report 2000.

[28] Gowdu B.J., Balasubramanian R. (1993). Biocontrol potential of rust of peanut by *Acremonium obclavatum*. Can. J. Bot..

[29] Manjula K., Kishore G.K., Podile A.R. (2004). Whole cells of *Bacillus subtilis* AF 1 proved more effective than cell-free and chitinase-based formulations in biological control of citrus fruit rot and peanut rust. Can. J. Microbiol..

[30] Podile A.R., Kishore G.K., Gnanamanickam S.S. (2002). Biological control of peanut diseases. Biological Control of Crop Diseases.

[31] Gowda M.V.C., Motagi B.N., Naidu G.K., Diddimani S.B., Sheshagiri R. (2002). GPBD 4: A Spanish bunch peanut genotype resistant to rust and late leaf spot. Int. Arachis Newsl..

[32] Liao B., Lei Y. (2004). A black seed coat peanut cultivar Zhonghua 9 released in China. Int. Arachis Newsl..

[33] Reddy L.J., Nigma S.N., Moss J.P., Singh A.K., Subrahmanyam P., Mcdonald D., Reddy A.G.S. (1996). Registration of ICGV 86699 peanut germplasm line with multiple disease and insect resistance. Crop Sci..

[34] Singh A.K., Dwivedi S.L., Pande S., Moss J.P. (2003). Registration of rust and late leaf spot resistant peanut germplasm lines. Crop Sci..

[35] Mace E.S., Phong D.T., Upadhyaya H.D., Chandra S., Crouch J.H. (2006). SSR analysis of cultivated peanut (*Arachis hypogaea* L.) germplasm resistant to rust and late leaf spot diseases. Euphytica.

[36] Stalker H. (1997). Peanut (*Arachis hypogaea* L.). Field Crop Res..

[37] Pasupuleti J., Nigam S., Pandey M.K., Nagesh P., Varshney R.K. (2013). Peanut improvement: Use of genetic and genomic tools. Front. Plant Sci..

[38] Bromfield K.R., Bailey W.K. (1972). Inheritance of resistance to *Puccinia arachidis* in peanut. Phytopathology.

[39] Nigam S.N., Dwivedi S.L., Gibbons R.W. (1980). Grounnut Breeding at ICRISAT.

[40] Kishore B. (1981). Rust Inheritance Studies in Peanut (*Arachls hypogaea* L.). Master’s Thesis.

[41] Tiwari S.P., Ghewande M.P., Misra D.P. (1984). Inheritance of resistance to rust and late leaf spot in peanut (*Arachis hypogaea* L.). J. Cytol. Genet..

[42] Paramasivam K., Jayasekhar M., Rajasekharan R., Veerabadhiran P. (1990). Inheritance of rust resistance in peanut (*Arachis hypogaea* L.). Madras Agric. J..

[43] Joel A., Sumathi P., Raveendran T. (2006). Genetics of rust (*Puccinia arachidis* Speg.) and its association with rust related traits in peanut (*Arachis hypogaea* L.). Plant Arch..

[44] Singh A.K., Moss J.P. (1984). Utilization of wild relatives in the genetic improvement of *Arachis hypogaea* L.. Theor. Appl. Genet..

[45] Wynne J.C., Beute M.K., Nigam S.N. (1991). Breeding for disease resistance in peanut (*Arachis hypogaea* L.). Ann. Rev. Phytopathol..

[46] Hayman B.I. (1958). The separation of epistatic from additive and dominance variation in generation means. Heredity.

[47] Ghewande M.P., Upadhyay R.K., Mukherji K.G., Chamola B.P., Dubey O.P. (2009). Rust of peanut-an overview. Integrated Pest and Disease Management.

[48] Subrahmanyam P., Moss J.P., Rao V.R. (1983). Resistance to peanut rust in wild *Arachis* species. Plant Dis..

[49] Pande S., Rao N.J. (2001). Resistance of wild *Arachis* species to late leaf spot and rust in greenhouse trials. Plant Dis..

[50] Fávero A.P., Moraes S.A., Garcia A.A.F., Valls J.F.M., Vello N.A. (2009). Characterization of rust, early and late leaf spot resistance in wild and cultivated peanut germplasm. Sci. Agric..

[51] Leal-Bertioli S.C.M., Cavalcante U., Cavalcante E.G., Ballén-Taborda C., Shirasawa K., Guimarães P.M., Jackson S.A., Bertioli D.J., Moretzsohn M.C. (2015). Identification of QTLs for rust resistance in the peanut wild species *Arachis magna* and the development of KASP markers for marker assisted selection. G3 Genes Genomes Genet..

[52] Liang X.Q., Li S.X., Li Y.C., Zhou G.Y. (1999). Release of cultivars Yueyou 5 and Yueyou 79 in Guangdong, China. Int. Arachis Newsl..

[53] Mondal S., Badigannavar A.M., D’Souza S.F. (2012). Molecular tagging of a rust resistance gene in cultivated peanut (*Arachis hypogaea* L.) introgressed from *Arachis cardenasii*. Mol. Breed..

[54] Power I.L., Culbreath A.K., Tillman B.L. (2013). Characterization of resistance of peanut to *Puccinia arachidis*. Plant Health Prog..

[55] Kumari V., Gowda M., Tasiwal V., Pandey M.K., Bhat R.S., Mallikarjuna N., Upadhyaya H.D., Varshney R.K. (2014). Diversification of primary gene pool through introgression of resistance to foliar diseases from synthetic amphidiploids to cultivated peanut (*Arachis hypogaea* L.). Crop J..

[56] Gajjar K.N., Mishra G.P., Radhakrishnan T., Dodia S.M., Rathnakumar A.L., Kumar N., Kumar S., Dobaria J.R., Kumar A. (2014). Validation of SSR markers linked to the rust and late leaf spot diseases resistance in diverse peanut genotypes. Aust. J. Crop Sci..

[57] Upadhyaya H.D., Dwivedi S.L., Vadez V., Hamidou F., Singh S., Varshney R.K., Liao B. (2014). Multiple resistant and nutritionally dense germplasm identified from Mini Core Collection in peanut. Crop Sci..

[58] Sudini H., Upadhyaya H.D., Reddy S.V., Mangala U.N., Rathore A., Kumar K.V.K. (2015). Resistance to late leaf spot and rust diseases in ICRISAT’s mini core collection of peanut (*Arachis hypogaea* L.). Australas. Plant Pathol..

[59] Okori P., Monyo E.S., Okello D.K., Mponda O.K., Chamango A., Chintu J., Amane M., Mausch K., Monyo E.S., Varshney R.K. (2016). Enhancing peanut productivity and production in Eastern and Southern Africa. Seven Seasons of Learning and Engaging Smallholder Farmers in the Drought-Prone Areas of Sub-Saharan Africa and South Asia through Tropical Legumes, 2007–2014.

[60] Mondal S., Badigannavar A.M. (2018). Mapping of a dominant rust resistance gene revealed two R genes around the major Rust_QTL in cultivated peanut (*Arachis hypogaea* L.). Theor. Appl. Genet..

[61] Chaudhari S., Khare D., Patil S.C., Sundravadana S., Variath M.T., Sudini H.K., Manohar S.S., Bhat R.S., Pasupuleti J. (2019). Genotype × Environment Studies on Resistance to Late Leaf Spot and Rust in Genomic Selection Training Population of Peanut (*Arachis hypogaea* L.). Front. Plant Sci..

[62] Daudi H., Shimelis H., Mathew I., Oteng-Frimpong R., Ojiewo C., Varshney R.K. (2020). Genetic diversity and population structure of peanut (*Arachis hypogaea* L.) accessions using phenotypic traits and SSR markers: Implications for rust resistance breeding. Genet. Resour. Crop Evol..

[63] Daudi H., Shimelis H., Mathew I., Rathore A., Ojiewo C.O. (2021). Combining ability and gene action controlling rust resistance in peanut (*Arachis hypogaea* L.). Sci. Rep..

[64] Mondal S., Mohamed Shafi K., Raizada A., Song H., Badigannavar A.M., Sowdhamini R. (2022). Development of candidate gene-based markers and map-based cloning of a dominant rust resistance gene in cultivated peanut (*Arachis hypogaea* L.). Gene.

[65] Rajarathinam P., Palanisamy G.P.R., Narayana M., Alagirisamy M. (2023). Marker assisted backcross to introgress late leaf spot and rust resistance in peanut (*Arachis hypogaea* L.). Mol. Biol. Rep..

[66] Badigannavar A.M., Kale D.M., Mondal S., Murty G.S.S. (2005). Trombay peanut recombinants resistant to foliar diseases. Mutat. Breed. Newsl. Rev..

[67] Chuni L., Basu M.S., Hariprasanna K., Basu M.S., Singh N.B. (2014). Breeding peanut for biotic stresses. Peanut Research in India.

[68] Kalaimani S., Manoharan V., Varman P.V., Sridharan C.S., Thangavelu S., Dharmalingam V. (1996). An interspecific cross derivative-a new source of foliar disease resistance. Int. Arachis Newsl..

[69] Kolekar R.M., Sukruth M., Shirasawa K., Nadaf H.L., Motagi B.N., Lingaraju S., Patil P.V., Bhat R.S., Varshney R. (2017). Marker-assisted backcrossing to develop foliar disease-resistant genotypes in TMV 2 variety of peanut (*Arachis hypogaea* L.). Plant Breed..

[70] Shasidhar Y., Variatha M.T., Vishwakarma M.K., Manohar S.S., Gangurde S.S., Sriswathi M., Sudini H.K., Dobariya K.L., Bera S.K., Radhakrishnan T. (2020). Improvement of three popular Indian peanut varieties for foliar disease resistance and high oleic acid using SSR markers and SNP array in marker assisted backcrossing. Crop J..

[71] Yeri S.B., Bhat R.S. (2016). Development of late leaf spot and rust resistant backcross lines in JL 24 variety of peanut (*Arachis hypogaea* L.). Electron. J. Plant Breed..

[72] Nigam S.N., Reddy L., Subrahmanyam P., Reddy A.G.S., McDonald D., Gibbons R.W. (1992). Registration of ICGV 87157, An Elite Peanut Germplasm with Multiple Resistance to Diseases. Crop Sci..

[73] Deshmukh D.B., Marathi B., Sudini H.K., Variath M.T., Chaudhari S., Manohar S.S., Rani C.V.D., Pandey M.K., Pasupuleti J. (2020). Combining high oleic acid trait and resistance to late leaf spot and rust diseases in peanut (*Arachis hypogaea* L.). Front. Genet..

[74] Varshney R.K., Pandey M.K., Janila P., Nigam S.N., Sudini H., Gowda M.V.C., Sriswathi M., Radhakrishnan T., Manohar S.S., Nagesh P. (2014). Marker-assisted introgression of a QTL region to improve rust resistance in three elite and popular varieties of peanut (*Arachis hypogaea* L.). Theor. Appl. Genet..

[75] Reddy L.J., Nigam S.N., Rao R.C.N., Reddy N.S. (2001). Registration of ICGV 87354 peanut germplasm with drought tolerance and rust resistance. Crop Sci..

[76] Upadhyaya H.D., Nigam S.N., Reddy A.G.S. (2002). Registration of early maturing, rust, late leaf spot, and low temperature tolerant peanut germplasm line ICGV 92267. Crop Sci..

[77] Pasupuleti J., Pandey M.K., Manohar S.S., Variath M.T., Nallathambi P., Nadaf H.L., Sudini H., Varshney R.K., Singh R. (2016). Foliar fungal disease-resistant introgression lines of peanut (*Arachis hypogaea* L.) record higher pod and haulm yield in multilocation testing. Plant Breed..

[78] Subrahmanyam P., McDonald D., Waliyar F., Reddy L.J., Nigam S.N., Gibbons R.W., Rao V.R., Singh A.K., Pande S., Reddy P.M. (1995). Screening Methods and Sources of Resistance to Rust and Late Leaf Spot of Peanut.

[79] Cheng L., Guo J., Li W., Huang L., Luo H., Liu N., Zhou X., Chen W., Wang J., Lyu J. (2022). Novel genotypes and quantitative trait locus for rust resistance in peanut. Chin. J. Oil Crop Sci..

[80] Hammons R.O., Branch W.D., Bromfield K.R., Subrahmanyam P., Rao V.R., Nigam S.N., Gibbons R.W., Goldin E. (1982). Registration of Tifrust-13 peanut germplasm. Crop Sci..

[81] Hammons R.O., Branch W.D., Bromfield K.R., Subrahmanyam P., Rao V.R., Nigam S.N., Gibbons R.W. (1982). Registration of Tifrust-14 peanut germplasm (Reg. No. GP31). Crop Sci..

[82] Reddy L.J., Nigam S.N., Dwivedi S.L., Gibbons R.W. (1987). Breeding peanut cultivars resistant to rust (*Puccinia arachidis* Speg.). Peanut Rust Disease.

[83] Gowda M.V.C., Motagi B.N., Sheshagiri R., Naidu G.K., Rajendraprasad M.N. (2002). Mutant 28-2: A bold-seeded disease and pest resistant peanut genotype for Karnataka, India. Int. Arachis Newsl..

[84] Gorbet D.W., Norden A.J., Shokes F.M., Knauft D.A. (1987). Registration of Southern Runner peanut. Crop Sci..

[85] Reddy L.J., Nigam S.N., Subrahmanyam P., Reddy A.G.S., McDonald D., Gibbons R.W., Pentaiah V. (1993). Registration of “ICGV 86590” peanut cultivar. Crop Sci..

[86] Gorbet D.W., Shokes F.M. (2002). Registration of ‘C-99R’ peanut. Crop Sci..

[87] Gorbet D.W., Shokes F.M. (2002). Registration of ‘Florida MDR 98′ peanut. Crop Sci..

[88] Gorbet D.W. (2007). Registration of ‘Hull’ peanut. J. Plant Regist..

[89] Gorbet D.W., Tillman B.L. (2011). Registration of ‘York’ peanut. J. Plant Regist..

[90] Zhen Y., Chen X. (1996). Study on breeding of high-yield and rust-resistant peanut variety Shanyou 523. Guangdong Agric. Sci..

[91] Liang X.Q., Li Y.C., Li S.X., Zhou G.Y. (1999). Yueyou 223: A high yielding Chinese cultivar with good resistance to rust. Int. Arachis Newsl..

[92] Zheng Y., Chen X., Lin C. (1999). Breeding and production application of a peanut variety, Shanyou 27. Peanut Sci. Technol..

[93] Zheng Y., Chen X. (2000). Breeding of a new peanut variety Shanyou 71. Chin. J. Oil Crop Sci..

[94] Zhong R., Han Z., Jin H., Zhou C. (2003). Breeding and application of a new peanut variety Guihua 23 with high yield and multiple resistance. J. Peanut Sci..

[95] Wu J., Zhou S., Guan H., Liu S. (2003). A new little peanut variety Yuanza 9102. China Seed Ind..

[96] Chen A., Wu J., Chen H., Feng X., Yang W. (2003). Breeding of a new peanut variety Zhanyou 30 with high yield. J. Peanut Sci..

[97] Feng X., Chen A., Wu J., Chen H., Jiang R. (2004). Breeding of a new peanut variety Zhanyou 62. J. Peanut Sci..

[98] Zheng Y., Huang W., Chen X., Liang J., Qi G., Huang Y., Chen F. (2005). Multiple resistance, high yield and fine quality new peanut variety Shanyou 162. J. South. Agric..

[99] Fang X., Liao W., Xiao L., Zhou G., Li D., Cai S. (2007). A primary report on the introduction and demonstration of super high-yield peanut variety Yueyou 7. J. Peanut Sci..

[100] Li S., Zhou G., Liang X., Li S., Lin K., Hong Y., Chen X., Liu H. (2007). Breeding of a new peanut variety Yueyou 114 and its cultivation technologies. J. Peanut Sci..

[101] Wu J., Han T., Wang F. (2008). Shangyan 9658: A new peanut variety with high yield, multi-resistance and high oil. Crops.

[102] Chen J., Chen Y., Chen S., Luo W., Chen R., Zhuang M. (2008). Breeding of a new peanut variety Quanhua 7. J. Peanut Sci..

[103] Tang Z., Xu R., Lan X., Lin Y., Shi G. (2011). Breeding and cultivation of a new rust-resistant peanut variety, Fuhua 6. Fujian J. Agric. Sci..

[104] Chen Y., Zhang S., Li H., Xu Y., Zhang K. (2011). Breeding and cultivation of a new peanut variety, Shanyou 65. Guangdong Agric. Sci..

[105] Wang Y., Chen A., Feng X., Lin W., Chen X., Wang Y. (2011). Breeding of high-yield, good quality and disease-resistant new peanut variety Zhanyou 75 and its cultivation technologies. J. Peanut Sci..

[106] Li H., Zhang S., Chen X. (2011). Breeding of a new peanut variety Shanyou 12. Fujian Agric. Sci. Technol..

[107] Li J., Sun C., Yang Y. (2011). Characteristics and high-yield cultivation techniques of Kainongheihuasheng. Bull. Agric. Sci. Technol..

[108] Su Y., Huang T. (2012). A new peanut variety Yueyou 93. Fujian Agric..

[109] Chen R., Xu Y., Huang L., Zhang J., Li D., Cheng Y., Zeng L., Fang L., Lin Y., He S. (2014). Cultivation technology research and application of new peanut variety Zhongkaihua 1. Trop. Agric. Eng..

[110] Xu J., Zhang X., Tang F., Dong W., Zang X., Zhang Z., Han S., Qin L. (2014). Breeding of a new peanut variety Yuanza 9847 and its enlightenments. J. Henan Agric. Sci..

[111] Tang G.Y., Wang Y.Y., Liao B., Li D., Xia X.M., Duan N.X., Wang Z.Z., Gowda C.L.L., Nigam S.N., Johansen C., Renard C. (1996). Breeding a high-yielding and rust-resistant peanut cultivar, Zhonghua No. 4, and its application. Achieving High Peanut Yields.

[112] Liao B., Lei Y. (2007). Zhonghua No. 12 with high oil and red skin. Farmer’s Consult..

[113] Wu J., Xiao Z., Su Y., Ma Q., Liu S. (2005). Yuhua 15: A new peanut variety with early maturity, high yield and high oil. China Seed Ind..

[114] Su R., Wu J., Li K., Ma Q. (2010). A new peanut variety Shangyan 9938 and its high-yield cultivation technology. China Seed Ind..

[115] Mao R., Zhang J., Li W., Zhang W., Wu L., Chen J. (2003). A new high quality and high yield big peanut variety Huayu 18 and its high yield cultivation technology. J. Peanut Sci..

[116] Ye M. (1986). Yueyou 92: A new peanut variety with high resistance to bacterial wilt. Crops.

[117] Zhou G. (2001). An introduction of a new peanut variety-Guihua 21. Peanut Sci. Technol..

[118] Chen Y., Chen J., Zhuang M. (2003). Quanhua 327: A new peanut variety with high yield and high quality. Bull. Agric. Sci. Technol..

[119] Sugui F.P. (2004). A new peanut variety NSIC Pn 12 released in Philippines. Int. Arachis Newsl..

[120] Hou H., Liao B., Lei Y., Ren X., Wang S., Li D., Jiang H., Huang J., Chen B. (2007). Identification of AFLP markers for resistance to peanut rust. Chin. J. Oil Crop Sci..

[121] Mondal S., Badigannavar A.M., Murty G. (2007). RAPD markers linked to a rust resistance gene in cultivated peanut (*Arachis hypogaea* L.). Euphytica.

[122] Varma T.S.N., Dwivedi S.L., Pande S., Gowda M.V.C. (2005). SSR markers associated with resistance to rust (*Puccinia arachidis* Speg.) in peanut (*Arachis hypogaea* L.). SABRAO J. Breed. Genet..

[123] Khedikar Y.P., Gowda M.V.C., Sarvamangala C., Patgar K.V., Upadhyaya H.D., Varshney R.K. (2010). A QTL study on late leaf spot and rust revealed one major QTL for molecular breeding for rust resistance in peanut (*Arachis hypogaea* L.). Theor. Appl. Genet..

[124] Sujay V., Gowda M.V.C., Pandey M.K., Bhat R.S., Khedikar Y.P., Nadaf H.L., Gautami B., Sarvamangala C., Lingaraju S., Radhakrishan T. (2012). Quantitative trait locus analysis and construction of consensus genetic map for foliar disease resistance based on two recombinant inbred line populations in cultivated peanut (*Arachis hypogaea* L.). Mol. Breed..

[125] Kolekar R.M., Sujay V., Shirasawa K., Sukruth M., Khedikar Y.P., Gowda M.V.C., Pandey M.K., Varshney R.K., Bhat R.S. (2016). QTL mapping for late leaf spot and rust resistance using an improved genetic map and extensive phenotypic data on a recombinant inbred line population in peanut (*Arachis hypogaea* L.). Euphytica.

[126] Pandey M.K., Khan A.W., Singh V.K., Vishwakarma M.K., Shasidhar Y., Kumar V., Garg V., Bhat R.S., Chitikineni A., Varshney R.K. (2017). QTL-seq approach identified genomic regions and diagnostic markers for rust and late leaf spot resistance in groundnut (*Arachis hypogaea* L.). Plant Biotechnol. J..

[127] Shirasawa K., Bhat R.S., Khedikar Y.P., Sujay V., Kolekar R.M., Yeri S.B., Sukruth M., Cholin S., Asha B., Pandey M.K. (2018). Sequencing analysis of genetic loci for resistance for late leaf spot and rust in peanut (*Arachis hypogaea* L.). Front. Plant Sci..

[128] Ahmad S., Nawade B., Sangh C., Mishra G.P., Bosamia T.C.T.R., Kumar N., Dobaria J.R., Gajera H.P. (2020). Identification of novel QTLs for late leaf spot resistance and validation of a major rust QTL in peanut (*Arachis hypogaea* L.). 3 Biotech.

[129] Bertioli D.J., Cannon S.B., Froenicke L., Huang G.D., Farmer A.D., Cannon E.K.S., Liu X., Gao D.Y., Clevenger J., Dash S. (2016). The genome sequences of *Arachis duranensis* and *Arachis ipaensis*, the diploid ancestors of cultivated peanut. Nat. Genet..

[130] Zhuang W.J., Chen H., Yang M., Wang J.P., Pandey M.K., Zhang C., Chang W.C., Zhang L.S., Zhang X.T., Tang R.H. (2019). The genome of cultivated peanut provides insight into legume karyotypes, polyploid evolution and crop domestication. Nat. Genet..

